# Healthcare Professionals' Experiences with Rehabilitation Practices for Patients with Cognitive Impairment after Stroke in North Norway: A Qualitative Study

**DOI:** 10.1155/2022/8089862

**Published:** 2022-09-10

**Authors:** Anniken Bogstrand, Astrid Gramstad, Audny Anke, Henriette Holm Stabel, Cathrine Arntzen

**Affiliations:** ^1^Department of Health and Care Sciences, Faculty of Health Sciences, UiT-The Arctic University of Norway, 9037 Tromsø, Norway; ^2^Centre for Care Research, North, Department of Health and Care Sciences, Faculty of Health Sciences, UiT-The Arctic University of Norway, 9037 Tromsø, Norway; ^3^Department of Rehabilitation, University Hospital of North Norway, 9038 Tromsø, Norway; ^4^Department of Clinical Medicine, Faculty of Health Sciences, UiT-The Arctic University of Norway, 9037 Tromsø, Norway; ^5^Research Centre for Habilitation and Rehabilitation Model and Services (CHARM), University of Oslo, Oslo, Norway; ^6^Hammel Neurorehabilitation Centre and University Research Clinic, University of Aarhus, Voldbyvej 15, DK-8450 Hammel, Denmark

## Abstract

**Methods:**

A focus group interview with clinicians, coordinators, and leaders involved in stroke survivors' rehabilitation trajectories was conducted. The group consisted of a strategic selection of participants with diverse professional backgrounds from specialist and primary healthcare services. The transcribed material was analyzed thematically using systematic text condensation based on an inductive, interpretive approach.

**Results:**

We found that patients with mild and moderate cognitive impairment after stroke were characterized as a neglected group in rehabilitation services and that neglect was related to both structural and professional issues. First, neglect seemed partly related to the availability of existing healthcare services, which mainly followed up on physical challenges after stroke. Second, cognitive rehabilitation seemed to be less prioritized than other health services, and the established interdisciplinary municipality teams did not seem prepared to follow-up on cognitive issues. Finally, at a professional level, the study reveals the need for building competence in cognitive rehabilitation and having services available in the long run.

**Conclusion:**

The study demonstrates the need to increase knowledge concerning cognitive rehabilitation and how rehabilitation trajectories and services should be organized to fulfil stroke survivors' and carers' long-term needs.

## 1. Introduction

Cognitive impairment is a frequent consequence following stroke. Nearly half of stroke survivors have been reported to display a varied level of poststroke cognitive impairment (PSCI) during the first year [1]. Cognitive impairments such as aphasia, apraxia, neglect, visuospatial difficulties, and anosognosia, as well as reduced attention, psychomotor pace, concentration, and memory, and difficulties with executive functions, are common [2]. These impairments can cause multiple challenges in regard to managing everyday life activities [3], such as the ability to live independently at home [4, 5], the capacity for work [6], and the ability to maintain interpersonal relationships [7]. Even mild cognitive impairments can have a profound impact on quality of life [8–10].

Many stroke survivors and their next of kin need help and support from health professionals, placing special demands for appropriate services in different phases of the rehabilitation process [11, 12]. A growing number of studies have shown that cognitive rehabilitation can lead to clinically significant improvements even years after stroke [13, 14], increase independence in daily life and promote integration back to society [15]. Studies have shown that recovery after stroke is a long-term process in which the stroke survivor gradually modifies new bodily habits, repositions participation in everyday life, and renews his or her sense of self-identity [16, 17]. Another study demonstrated how changes in activities and relations affect established positions within the family, and that rehabilitation must address these challenges [18]. International studies have identified considerable variation in and lack of management for patients with cognitive impairments and their families, especially in the long term [19–22]. Higher levels of unmet needs have been reported among stroke survivors with PSCI than in patients with physical impairments in the long-term community reintegration phase [22].

In Norway, rehabilitation usually takes place in hospitals [23], where most acute stroke patients (95%) are admitted to stroke units [24]. A recent study from the northern part of Norway showed that approximately 40% of participants received further inpatient treatment at rehabilitation units, 17% received community-based rehabilitation, and 43% received no rehabilitation after discharge from stroke units [25]. However, this study revealed little about the content of professional support for community-based services or which kind of patients' needs these services applied to. A qualitative study investigating the content of one-year follow-up in the same area indicated that community-based services for stroke survivors mainly provided support regarding physical functioning, which did not necessarily meet patients' needs [26]. Family support and rehabilitation for managing daily life with cognitive and psychosocial challenges have been scarcely addressed [26, 27].

It is well known that patients with mild or moderate impairment after stroke in most cases benefit the most from rehabilitation in their own environment, if possible, combined with day-rehabilitation services [13]. Access to stimulating activities at home organized by coordinated multidisciplinary teams may reduce long-term dependency and admission to institutional care, as well as reduce the length of hospital stay [28]. However, Norwegian municipalities report difficulties offering multidisciplinary person-centred rehabilitation services after discharge home [29]. Access to healthcare staff and lack of rehabilitation expertise is particularly prominent in rural areas [30].

The aim of this study is to investigate how multidisciplinary health professionals experience long-term services provided to patients with mild and moderate PSCI in a North Norwegian context. We explore how organizational and individual structures contribute to shaping the service provision for this specific patient group in a given geographical area.

## 2. Materials and Methods

### 2.1. Study Design

This research employed a qualitative design, which allowed for an in-depth exploration of cognitive rehabilitation practices after stroke. To answer the research question, we conducted a multidisciplinary focus group interview with both specialist and community healthcare service providers. Focus group interview was considered suitable because it facilitates interaction that may elicit experiences and ideas and elaborate research participants' perspectives through debate within the group [31, 32]. The research method has been increasingly recommended as useful for obtaining views concerning health and health services from users, caregivers, and service providers [33]. The study adheres to the Consolidated Criteria for Reporting Qualitative Research (COREQ) guidelines [34], to ensure the quality of the research process and its reporting.

### 2.2. Participants

A purposeful sampling strategy was used [35], through which we strived to recruit a broad selection of participants from both in-hospital specialist healthcare and out-patient municipality services who encounter stroke survivors with PSCI. The participants were recruited following telephone contact to managers in the organizations of a university hospital in North Norway and at municipal departments. Everyone asked was willing to participate in the study. Informed consent was returned to the first author (AB). Seven multidisciplinary professionals, six women and one man, were included in the study. All participants had broad experience within the field of neurorehabilitation, ranging from five to twenty-four years. The clinicians (a nurse, a PT, two OTs), two coordinators (from a university hospital and a municipality team), and a leader of a municipal rehabilitation unit worked within coordinating services, different interdisciplinary teams, and a reablement service program.

Three of the participants worked at a patient-centred healthcare team for elderly individuals with complex and chronic diseases, aiming at strengthening transitions between hospital and municipalities. One participant represented an ambulatory rehabilitation team providing prolonged advisory follow-up from hospital to home communities to patients of working age with complex, long-term rehabilitation needs. One participant represented a municipal-based interdisciplinary reablement team that offer time-limited home training to patients with mild rehabilitation needs. Their aim is to provide support in reoccupying activities that are important to people in their everyday life. Also, one therapist worked with community-based, institutional rehabilitation that provided interdisciplinary rehabilitation, either prior to, -after, -or instead of hospitalization. Last, one participant worked administratively at a coordinating municipal team aiming to coordinate the flow in services between specialist and primary healthcare to adult patients with somatic, complex rehabilitation needs.

### 2.3. Interview Procedure

The interview guide was jointly developed in cooperation with all authors. Moreover, in the preparation phase, the content of the interviews in the project were discussed with a reference group that consisted of patients and next of kin from a stroke organization, which aided in developing the guide. The main theme for the focus group was discussions about the multidisciplinary professionals' experiences concerning service support for patients with mild and moderate cognitive impairments. Key points were patients' needs and changing needs, professional content and support, collaboration, and challenges and ideas for improvements.

The focus group interview took place in a meeting room at the first author's workplace. The first author (AB) led the interview, presenting topics for discussion and encouraging the participants to share their experiences and discuss within the group. The interviewer and PhD candidate had a background as an occupational therapist with primary knowledge and experience working with neurorehabilitation. The coauthor (AG) followed up with supplementary questions and took notes to help summarize the interview. The focus group interview lasted two hours and was audio-recorded and subsequently transcribed verbatim by the first author.

### 2.4. Data Analysis

The material was analysed using systematic text condensation (STC) [36], which is inspired by Georgi's psychological phenomenological analysis and consists of four steps. The first step involved reading the transcribed data and listening to the audiotaped recordings to obtain an overview of the first-impression themes. Preliminary written notes of the overall impression and themes were shared and discussed initially with one of the coauthors AG. In the second step, the first author identified and coded meaning units in the material about the participants' experiences with cognitive rehabilitation services. In the third step, the meaning units derived from the code groups in the previous stage were abstracted into condensed units. At this stage, the data were reduced to a decontextualized selection of meaning units that were sorted across the individual participant contributions according to Malterud's approach (p. 799). In the final step, the condensed units were recontextualized and written as descriptions. The analysis was supported by utilizing the qualitative data analysis software program NVivo 12 plus (QSR international) [37], which was useful in the process of coding and categorizing the data. The first author AB during the STC process and validated by two of the coauthors AG and CA until agreement was reached. The final descriptions were a result of a hermeneutical process moving back and forth between the transcripts, temporary findings, existing research, and relevant literature to ensure that the recontextualized descriptions were grounded in the empirical data. Two of the authors AA and HHS contributed to the later stage of the analysis process, scrutinizing the themes and ensuring that they were concise and clear.

### 2.5. Ethical Considerations

The authors received approval for the study from the Norwegian Centre for Research Data (reference number 60366), according to personal data protection. The multidisciplinary professionals signed an informed consent form to participate and were informed that they could withdraw at a given time without specifying the reason. The data were treated confidentially, and information about the participants was anonymized and presented with caution to avoid being traceable to individual contributions.

## 3. Results

Through the thematic analysis, we identified three main themes. The initial two reflect experiences of services available and healthcare priorities at a structural level in relation to patients with PSCI. The last theme concerns the experiences of health professionals' needs in terms of building competence and providing access to cognitive rehabilitation competencies, where available, in the long-term follow-up (see Table 1).

An overall finding was that the participants described patients with mild and moderate cognitive impairments after stroke as being a neglected patient group throughout the rehabilitation trajectory. According to the participants, the inattention to this group during the early inpatient stay could be due to a lack of investigation and therefore a missed opportunity to reveal the patient's cognitive challenges. In the phase of transitioning home, the inattention could be due to not identifying patterns in cognitive consequences over time and discussing a follow-up plan. The negligence in the long run could be related to not having services available in the municipality when cognitive impairments make everyday life for both the stroke survivor and their family members difficult. Overall, patients with mild and moderate PSCI can be characterized as a neglected patient group because it seems that the system is not capable of identifying their needs and having trained personnel available for cognitive rehabilitation, particularly in the long-term rehabilitation trajectory.

### 3.1. Patients with Mild and Moderate PSCI Do Not Fit into the Traditional Services

A recurring issue in the material was that the participants described that this group of patients did not fit into the traditional rehabilitation services. One participant explained,

"If the main concern is cognition, then the experience is that there are often challenges because who is there to follow-up, then? It has something to do with attaching it [cognitive rehabilitation needs] to some services, because they do not belong in any of the traditional services."

The participants used the term “traditional services” when referring to the healthcare services that were usually provided at hospitals, community centres, and outpatient clinics to patients in need of rehabilitation in general. Common features were that follow-up begins as early as possible, that health services provide intensive follow-up on specific functional impairments and over a limited period. When talking about the traditional services in the municipalities, the participants often referred to speech therapy, OT, PT, and different interdisciplinary teams. However, the participants experienced that the patients in this group “do not fit into the ways in which the traditional services are organized.”

Several participants described that from the early inpatient rehabilitation phase, a main concern often involved providing intensive follow-up on physical functioning, compared to assessing and supporting cognitive and psychosocial needs. However, different viewpoints were discussed. One participant expressed that in regard to stroke patients in general, cognitive needs and recommendations for further priorities seem readily described in patient reports and handover meetings. Another participant, however, expressed that for patients with especially mild and moderate PSCI, cognitive concerns were “perhaps not most commonly described.” The consequences when cognitive and psychosocial issues were not being identified and addressed by professionals initially were discussed. One participant said,

"… we know that many have been discharged when they are physically fine. Maybe there is a mild impairment. But… you do not know for certain, and… maybe those who know him well, are starting to notice it after some weeks, and… where they are sent home after a few days at the hospital. Without a further plan, then."

However, when cognitive impairments were assessed and followed up and when referrals were made before discharge, the participants experienced that the services in the municipalities were not adjusted to the patients' cognitive needs for support. A lack of services that could address these needs in the municipality was one aspect. One participant said, “Like we've talked about, having an overview at a municipal level, we don't have sufficient health care services available to this patient group.” For example, several participants described follow-up in returning to work life being “somewhat neglected,” as such follow-up requires close cooperation between the patients, interdisciplinary health professionals, and different sectoral services, although no one was appointed to follow-up in this domain. Several participants also talked about not having designated coordinators or health professionals appointed to a liaison role for stroke service provision in the community that could prioritize cognitive rehabilitation. A participant who worked in one of the interdisciplinary teams described overall challenges with the availability of health professionals who could assist patients with cognitive concerns:

"…it depends on having someone to cooperate with, whether they [the municipalities] have someone they can give that task to… if there are any personnel available, if they [the patients] do not need anything else. If home care service is there already or there are occupational therapists available, if they are a part of follow-up or who receives counselling. Then, there is someone for our part at least, who we can collaborate with."

The participants described that to support recovery after stroke, it was essential to offer early, intensive multiprofessional rehabilitating support. Simultaneously, they discussed how in many circumstances cognitive challenges often first become apparent for patients and relatives after living at home for some time, further highlighting how cognitive rehabilitation requires quite different organization and content for follow-up. One participant described, “Cognitive rehabilitation takes time. In a way, you need the services after you have finished the physical part, or you need help with care. You still need time... for the cognitive rehabilitation.” Another participant said,

"Many fall, as you mentioned, in between two chairs. As the cognitive impairments become perhaps more visible…then there is no one there guiding them further. They sort of do not fit into any boxes in the municipalities."

The participants further discussed the importance of carrying out cognitive rehabilitation in settings and through activities that are familiar and important to the patient. One participant expressed, “The world outside the hospital could be totally different for the patient. Their focus and needs may have changed completely.” Except for reablement teams that provided home training, traditional rehabilitation services rarely took place in the patients' natural everyday life surroundings. One participant said, “That's where it's missing; we do have day services, but we must have services available for those who need it at home.”

In summary, the descriptions revealed a gap between how the services were organized and what patients' cognitive rehabilitation needs were. Patients with mild and moderate PSCI were reported to have needs that required following another timeline and possessing another understanding of their recovery process. Overall, the participants acknowledged that this patient group was being overlooked in different stages throughout the rehabilitation trajectory and increasingly overtime.

### 3.2. Patients with Mild and Moderate PSCI are Being Deprioritized in Several Ways

Priorities in healthcare services also seem to influence why this patient group appears to be systematically neglected. An interesting finding was that cognitive rehabilitation may suffer a triple burden: first, rehabilitation does not seem to be a priority service compared to other service needs; second, cognitive rehabilitation seems to be a deprioritized area within the rehabilitation field; and third, established interdisciplinary teams in the municipalities do not seem to be attuned to supporting the needs of this patient group.

The quote below illustrates that rehabilitation in general is not given a very high priority and seems to be a nonpriority service compared with supporting care and nursing needs.

"Rehabilitation is… far out in the rank of priorities. Number one is what we must do first, and that is to make sure the patient receives, for instance, nursing and care, according to their needs. That is a high level of attention. While the rehabilitation part – it can wait."

Another participant described how cognitive rehabilitation appeared to not be prioritized within the rehabilitation field: “There are few municipalities that have someone dedicated to work with cognitive functioning.” Several participants described excessive attention to physical functioning in both specialist and primary healthcare services. A “bodily focus” was apparent in that the visible, physical, and impaired body was reported to be initially at the forefront of health personnel's attention. It was also the physical body that patients first became aware of and requested help for recovery. One participant said, “There is often a bodily focus, and then the cognitive part comes… somewhat on the sideline.” Attention to bodily functioning from an early, postacute phase was reported to be necessary to minimize long-term medical and physical consequences. Even so, this was reflected as having implications for the rehabilitation process in the sense that health professionals were not always prompted to look for, attend to, and work with cognitive functioning.

Furthermore, the participants described their engagement in different interdisciplinary teams that were aimed at follow-up either in the transition between hospital and home or that were established in some of the municipalities. For example, clinicians who worked at a patient-centred healthcare team stated that they addressed overall issues related to physical functioning and facility adjustments in the home environment in general, although they rarely attended to rehabilitation towards stroke patients. One participant said, “…it is not like specifically, that we come in and rehabilitate someone. At least not cognitively.” Another participant who worked in a reablement service described providing intensive rehabilitation for a limited period, elaborating as follows:

"…it is a general rehabilitation form, where we should go in for four weeks and it should be intensive, at least five days a week. And we do have quite a few stroke patients, but then, there are some that need longer follow-up than we can provide. And they fall a bit “in between chairs.”"

The participants acknowledged that the teams were not tailored to meet these patients' long-term needs and that cognitive rehabilitation was not considered a prioritized area. The exception was an ambulatory rehabilitation team. A participant engaged in this team described that many patients and next of kin who attended their self-management program one year after a mild or moderate stroke reported no prior follow-up after discharge from the hospital in terms of addressing cognitive impairments. In general, the participants described a lack of a formal structured system available in each municipality for stroke service provision that prioritized cognitive rehabilitation.

"We need to have a professional emphasis on cognitive rehabilitation in the municipalities. In a larger municipality, you can have an interdisciplinary team, right? That is harder in smaller municipalities, where multiple tasks must be done at once, though... that there is someone who gets good at it and that you have some guidelines on what to do."

All in all, a healthcare system ranking physical functioning and physical needs first and foremost was portrayed. Moreover, rehabilitation in general, especially cognitive rehabilitation, was reported to be deprioritized among other healthcare service needs. Although several different interdisciplinary teams were established, cognitive rehabilitation was not a prioritized area. Thus, cognitive rehabilitation had to break through several layers of barriers to address these needs when necessary.

### 3.3. Developing Professional Competencies and Utilizing Competence That are Present

The deprioritization of cognitive rehabilitation at a structural level seemed to have a direct impact on professional support and how health services were put to practice. One participant said, “It's about having it [cognitive rehabilitation] as a devoted part, that it is something one should work with and become good at – I mean, in terms of competence.”

The participants described a need for developing competence in cognitive rehabilitation and for having services available. For instance, an absence of interdisciplinary rehabilitation teams and OTs with diagnosis-specific competence were described, especially in some of the smaller municipalities. While some municipalities had health professionals who worked with this patient group, limited time for competence building was reported:

"Occupational therapists in the municipalities do follow-up over time. However, we have challenges when it comes to waiting lists. Nevertheless, that is where patients are referred because that's the right health service. But they have had… a lack of time, because of the pressure on… it becomes a great deal of fixing assistive devices and less time left for building competence or working on rehabilitation."

The participants also expressed a need for increased resources and time for competence development in home care services. However, although playing an important part in different patient group rehabilitation trajectories, home care was reported as being more often provided to stroke survivors if they had physical impairment as well. One of the clinicians elaborated as follows:

"There is no room for home care services to follow-up interventions they [stroke survivors] have received on these skills at the health centre. I think they could reach a higher functional level if they were given more time… for cognitive rehabilitation at home."

In addition, the participants highlighted how addressing patients' cognitive rehabilitation needs also seemed to rely on health professionals' opportunities to utilize their professional competence. One participant expressed that OTs and others had competence working with cognitive rehabilitation but that “they do not get in position to use it.” Another indicated that there are health professionals that “want, can, and work with cognitive matters in the municipalities.” Nevertheless, few had cognitive rehabilitation as a dedicated and explicit task in their job descriptions. Consequently, other requested assignments were prioritized.

Last, competencies that were available, although less known and therefore not sufficiently utilized, were discussed. For example, several participants described a lack of awareness of day centre services and therapists who had competence in cognitive rehabilitation and could follow-up patients of working age overtime. While every municipality should have a coordinating unit to turn to for information, it seemed undefined who was to obtain and provide information and when about the poststroke situation and competence available to support patients' recovery.

"… so, the problem lies with us, not the ones [the patients or caregivers] trying to get in hold of [services]. I mean we are “off the hook.” Instead, we are the ones that should provide information about the professional support available."

All things considered, professional competencies in general appeared not adjusted to meet these patients' needs in the long term. Although some services that provide relevant competence were described, health professionals with cognitive rehabilitation skills in this study region were reported to either not be in a position to use inherent resources or not be sufficiently utilized.

## 4. Discussion

The aim of this study was to explore multidisciplinary health professionals' experiences with rehabilitation trajectories and factors affecting healthcare services to patients with PCSI in a North Norwegian context. The results show that the participants found that the long-term rehabilitation services after discharge from hospital in general were not adjusted to patients' most prominent needs. Recurring issues in the empirical material were that patients with mild and moderate PSCI were at risk of receiving few services, receiving follow-ups that were not tailored their needs or being left with no services at all.

Although cognitive impairments in many cases have a profound impact on everyday life for patients and their families, there is a lack of services that address cognitive rehabilitation needs in municipalities. However, our study showed that if services were available, emphasis was not given to *when* during the stroke survivors' trajectories follow-up was needed. A review based on self-reported cognitive disorders summarized cognitive difficulties as a prominent phenomenon after stroke, where the consequences seem to intensify overtime through increasing demands for activity and participation [38]. Thus, cognitive rehabilitation services are often needed beyond the acute and early stages of rehabilitation. However, the findings of the current study show that health services are not adjusted to follow stroke survivors' recovery timeline. In Norway, an increased part of patients' stroke trajectories has been assigned to the municipalities because of the coordination reform [39] that was implemented in 2012. The reform determined specific responsibilities between specialist and primary healthcare. The aim was to support a higher level of coordinated and integrated health services, reduced in-hospital rehabilitation, and widened responsibilities for care and treatment of patients and users in the municipality [40]. These structural changes could contribute to services adapted to the need for long-term follow-up for this group, but in a North Norwegian context, this seems difficult to achieve for this patient group. Local health authorities are the key players responsible for providing services to adapt the home situation after discharge from hospitals. Nevertheless, the optimal organization of long-term follow-up of particularly patients with cognitive impairment and speech and language deficits has been scarcely addressed [23, 39]. Optimal follow-up for this patient group will probably require closer and more mutual cooperation across sectors and service levels.

The results further reveal that training is rarely given in patients' own environments. Initially, during postacute inpatient rehabilitation, interdisciplinary professionals may adjust interventions that align with the patient's stage of recovery. At the same time, these interventions are not performed in ideal surroundings, whereby the activities are out of context and apart from the familiar environment. After discharge to home, different needs often occur. These needs may be reflected in more advanced instrumental activities of daily living, where access to the patients' everyday life and habitual surroundings is called for [41]. Nevertheless, except for reablement services, interventions in this study region are mainly instigated at institutions. Previous research supports that therapy-based rehabilitation services targeted towards stroke patients living at home promote independence in personal activities of daily living, a higher likelihood of maintaining relearned abilities and reintegration into society [42]. Recommended follow-up can be challenging in more dispersed rural municipalities, where economic resources, professional competence, and interaction challenges between service levels differ [23].

In line with previous research [21, 22, 26], the results show that physical recovery and medical needs were prioritized in the delivery of rehabilitation services, while cognitive matters were described as “left somewhat on the sideline.” This finding is consistent with previous research underpinning how the overall priorities within rehabilitation historically have been focused on improving physical functioning [9]. Another possible explanation, consistent with former studies [20, 21, 43], is that the healthcare services to this patient group are influenced by the nature of residual, hidden needs. Thus, since physical impairments are often dominant symptoms, mild and moderate cognitive impairments are not necessarily picked up and talked about, either by clinicians or patients, striving for physical recovery initially. Lack of insight into patients' own cognitive problems and stigma are also aspects that may reinforce the invisibility of the problem [44]. Another contributing factor that may partly explain why PSCIs are overlooked in healthcare is challenges related to coinciding symptoms. A recent Canadian study [45] summarized the prevalence of cognitive impairment, along with fatigue and depression, following stroke, with approximately 20-50% of patients being affected by at least one of the three conditions. Although these conditions present challenges that may impede recovery and result in poor functional outcomes and decreased quality of life [46], they are often neglected domains of care. This could be related to symptoms that often overlap, which may contribute to escalating complexity regarding diagnosis and further proper treatment. Hence, the importance of cognitive screening and assessment practices, as well as adequate interventions that are coordinated and integrated into existing stroke guidelines, is emphasized, both in acute rehabilitation in stroke units, during discharge and in the subacute stages of care [45]. In the current study, no standardized cognitive assessments were reported to be utilized by primary care health professionals as part of the rehabilitation support.

Furthermore, our study found that there is often a lack of multidisciplinary teams in municipalities: where teams are established, they are generic in nature, and PSCIs are seldom part of the teams' assignment. Within the field of multidisciplinary healthcare, a systematic review pointed out that to deliver effective care across stroke trajectories, collaborating teams, especially when adopting an interdisciplinary approach, are a key contributor to quality in stroke services [47]. However, a comparative analysis of rehabilitation services in municipalities in one region in North Norway and one region in Denmark revealed considerable differences in abilities to support stroke survivors in adjustment, learning, and change, depending on, e.g., access to coordinated, qualified multidisciplinary teams, which were described as lacking in the North Norwegian region [27]. Consequently, and as the current study revealed, increased interdisciplinary collaboration requires access to multidisciplinary teams with stroke-specific competence and available services from professionals with competence in cognitive rehabilitation, such as occupational therapists and neuropsychologists. Service development is needed to create stronger links between specialist and primary healthcare and within municipalities.

The participants acknowledged various factors impacting health professionals' competence and discussed how clinical competencies were put into practice. Some of the obstacles in providing long-term management were described as a lack of resources in terms of interdisciplinary stroke-specific teams, coordinators, and health professionals addressing cognitive impairments and to overcome the workload for competence development. The participants reported no designated public health professionals specialized in supporting cognitive rehabilitation in the municipalities, except services at a day centre, which were reported as not being sufficiently utilized. In rural areas and small municipalities, the availability of professionals with stroke specific competence are difficult to obtain [26]. Ensuring good services where people live will require more interdisciplinary, cross-level, and cross-sectoral collaboration, as well as more digitally supported services.

The Norwegian Stroke Guidelines recommend that stroke survivors with mild to moderate impairments are discharged directly from hospital with an equivalent program of rehabilitation provided in their own home settings [23], as it is the best documented concept to this patient group [13, 28]. A systematic review on early supported discharge found that well-informed transitions, follow-up by a specialized multidisciplinary team, and close cooperation between hospitals and local healthcare services are valuable for people with mild and moderate impairments following stroke [28]. However, this portfolio is based on research in cities and suburbs, and the apparent benefits are largely derived from services provided by coordinated, early supported discharge teams. The role of these services has not been adequately addressed in poorer healthcare settings and rural district communities [28]. In the northern part of Norway, early supported discharge is implemented to a small degree. Overall, professional support and competencies need adjustment to regional differences, both in the transition home phase and in the long-term follow-up.

As discussed by Heiberg et al. [25], in the broad geographic rural North Norwegian region, available specialized subacute inpatient rehabilitation services are necessary. Parallel with building systems for improved transitions from stroke units and rehabilitation units to home, improved follow-up of persons with PSCI in the municipalities is further required. The findings in the current study demonstrate that such structural changes depend on increased capacity within the healthcare system and appropriate specialists to identify and address these matters.

## 5. Strengths and Limitations

Since our main focus was to explore follow-up in long-term trajectories, participants with experience with the transition home and further follow-up in the municipalities were included. Multidisciplinary professionals from both specialist and primary healthcare were strategically recruited for this study, representing a broad range of experiences and years working in the neurorehabilitation field. Thus, participants working in stroke units or inpatient rehabilitation units within specialist healthcare were not represented, which may have contributed to the exclusion of some perspectives in the material. The study was based on a small sample size. Although we strove for heterogeneity in the sample, coincidentally, only one man was represented due to the composition among employees at the different departments at the time. However, the group represented a diverse selection of professionals engaged in different interdisciplinary teams, coordination services, and a municipal rehabilitation unit. Their experiences added to our understanding of vital perspectives on different factors impacting healthcare services and stroke rehabilitation trajectories.

## 6. Concluding Remarks

The study demonstrates that stroke rehabilitation services focused on early, intensive time-limited support must be supplied with long-term support to help stroke survivors manage cognitive impairment in daily living. Greater efforts are needed to ensure a systematic development of the cognitive rehabilitation field in the North Norwegian area, where occupational therapists, speech therapists, and neuropsychologists in particular play an important role. The study shows that the availability of personnel and competence in cognitive impairments after stroke can be demanding in rural areas. To ensure good, personalized services, available ambulatory specialized rehabilitation services are necessary. Continued efforts are needed to further develop interdisciplinary, cross-sectoral, and digitally supported rehabilitation models to strengthen the rehabilitation service for this patient group.

A key policy priority should be to promote cognitive rehabilitation and further research addressing how long-term follow-up of these patients and their families should be organized to provide optimal rehabilitation services. This information can be used to further develop targeted interventions aimed at ensuring good follow-up for stroke patients with cognitive challenges in North Norway.

## Figures and Tables

**Table 1:** Overview of results.

Patients with PSCI do not fit into the traditional services	(i) Traditional service characteristics: early, intensive, time-limited, predominantly physical follow-up, and context neutral (ii) Rehabilitation needs: follow-up adjusted to recovery timeline, cognitive and psychosocial changes, and context (iii) Gap between patients' needs and existing services

Patients with PSCI are being deprioritized in several ways	(i) Rehabilitation priorities compared to other service needs (ii) Cognitive rehabilitation priorities within the rehabilitation field (iii) Cognitive rehabilitation within interdisciplinary teams

Developing professional competencies and utilizing competence that are present	(i) Investing in and building competence (ii) Getting in position to use competencies that are available (iii) Utilizing competencies that are less known or not sufficiently exploited

## Data Availability

The empirical material used to support the findings of this study are included within the article.
